# Association of Cerebrovascular Reactivity With 1-Year Imaging and Clinical Outcomes in Small Vessel Disease: An Observational Cohort Study

**DOI:** 10.1212/WNL.0000000000210008

**Published:** 2024-11-05

**Authors:** Emilie Sleight, Michael S. Stringer, Una Clancy, Carmen Arteaga-Reyes, Daniela Jaime Garcia, Angela C.C. Jochems, Stewart Wiseman, Maria Valdes Hernandez, Francesca M. Chappell, Fergus N. Doubal, Ian Marshall, Michael J. Thrippleton, Joanna M. Wardlaw

**Affiliations:** From the Centre for Clinical Brain Sciences (E.S., M.S.S., U.C., C.A.-R., D.J.G., A.C.C.J., S.W., M.V.H., F.M.C., F.N.D., I.M., M.T., J.M.W.) and UK Dementia Research Institute (E.S., M.S.S., U.C., C.A.-R., D.J.G., A.C.C.J., S.W., M.V.H., F.M.C., F.N.D., I.M., M.T., J.M.W.), University of Edinburgh, United Kingdom. Michael Thrippleton and Joanna Wardlaw are currently at Edinburgh Imaging Facility, Royal Infirmary of Edinburgh, University of Edinburgh, United Kingdom.

## Abstract

**Background and Objectives:**

In patients with cerebral small vessel disease (SVD), impaired cerebrovascular reactivity (CVR) is related to worse concurrent SVD burden, but less is known about cerebrovascular reactivity and long-term SVD lesion progression and clinical outcomes. We investigated associations between cerebrovascular reactivity and 1-year progression of SVD features and clinical outcomes.

**Methods:**

Between 2018 and 2021, we recruited patients from the Edinburgh/Lothian stroke services presenting with minor ischemic stroke and SVD features as part of the Mild Stroke Study 3, a prospective observational cohort study (ISRCTN 12113543). We acquired 3T brain MRI at baseline and 1 year. At baseline, we measured cerebrovascular reactivity to 6% inhaled CO_2_ in subcortical gray matter, normal-appearing white matter, and white matter hyperintensities (WMH). At baseline and 1 year, we quantified SVD MRI features, incident infarcts, assessed stroke severity (NIH Stroke Scale), recurrent stroke, functional outcome (modified Rankin Scale), and cognition (Montreal Cognitive Assessment). We performed linear and logistic regressions adjusted for age, sex, and vascular risk factors, reporting the regression coefficients and odds ratios with 95% CIs.

**Results:**

We recruited 208 patients of whom 163 (mean age and SD: 65.8 ± 11.2 years, 32% female) had adequate baseline CVR and completed the follow-up structural MRI. The median increase in WMH volume was 0.32 mL with (Q1, Q3) = (−0.48, 1.78) mL; 29% had a recurrent stroke or incident infarct on MRI. At 1 year, patients with lower baseline cerebrovascular reactivity in normal-appearing tissues had increased WMH (regression coefficient: B = −1.14 [−2.13, −0.14] log_10_ (%ICV) per %/mm Hg) and perivascular space volumes (B = −1.90 [−3.21, −0.60] log_10_ (%ROIV) per %/mm Hg), with a similar trend in WMH. CVR was not associated with clinical outcomes at 1 year.

**Discussion:**

Lower baseline cerebrovascular reactivity predicted an increase in WMH and perivascular space volumes after 1 year. CVR should be considered in SVD future research and intervention studies.

## Introduction

Cerebral small vessel disease (SVD) results from a disorder of the cerebral small vessels causing strokes^1^ and most vascular dementias.^2,3^ It is characterized by neuroimaging features observed with MRI—white matter hyperintensities (WMH), lacunes, microbleeds, visible perivascular space (PVS), and recent small subcortical infarcts.^4^ Currently, there is limited understanding of SVD pathophysiology, and no treatments are available.^5^ Identifying associations between vascular dysfunction and disease severity and progression may help develop treatments.^4^

Cerebrovascular reactivity (CVR) reflects the ability of cerebral blood vessels to dilate in response to a vasoactive stimulus such as CO_2_-enriched air. This type of stimulus has clearly been shown to affect the arterioles and the capillaries.^6^ By measuring change in blood oxygen level-dependent (BOLD) MRI signal in response to CO_2_-enriched air, CVR can be quantified^7,8^ and was shown to be impaired cross-sectionally in patients with SVD.^4,7,9^

In previous work, we analyzed how CVR in normal-appearing tissues and in WMH is associated cross-sectionally with SVD burden, stroke severity, functional outcome, and cognition.^10^ In agreement with other studies, we found that lower CVR was associated with higher WMH burden, more microbleeds, more lacunes, higher deep atrophy score, higher PVS score, and higher SVD score.^9,11^ Furthermore, one study reported that normal-appearing white matter (NAWM) tissues that progressed into WMH had lower CVR initially.^12^ However, the relation between impaired CVR and progression of SVD and related clinical outcomes has received little attention.^8,13^

As SVD is a disease of vascular origin, we aimed to assess the association between baseline CVR in subcortical gray matter (SGM), NAWM, and WMH; progression of neuroimaging (quantitative measurements of SVD burden at 3T); and clinical features (stroke severity and functional outcome, cognitive function, recurrent strokes/TIA, and new infarcts on brain MRI) in patients with stroke-related SVD. We hypothesized that lower baseline CVR would be associated with increasing SVD burden, increasing stroke severity, worse functional outcome, worsening cognitive function, and recurrent stroke or new infarcts over 1 year.

## Methods

We followed the STROBE reporting guidelines.^14^ The checklist can be found as a Supplementary Material.

### Patients

From August 2018 to June 2021, we recruited SVD patients with minor ischemic stroke (minor stroke defined as a modified NIH Stroke Scale [NIHSS] <8 and expected to be nondisabling, that is, modified Rankin Score [mRS] ≤2) presenting at Edinburgh/Lothian stroke services (Mild Stroke Study 3; ISRCTN 12113543).^15^ Stroke diagnosis was undertaken by specialist stroke physicians and neuroradiologists. We included patients with lacunar ischemic stroke (i.e., SVD-related stroke), because these patients reflect largely a clinical presentation of intrinsic SVD, and patients with cortical ischemic stroke representing primarily large artery atherothromboembolic stroke mechanisms. The latter have less SVD and thus contribute to a broader spectrum of SVD features allowing the generalization of the results beyond the lacunar stroke population, while having similar vascular risk factor (VRF) profiles and taking the same stroke prevention drugs which may affect vasoreactivity. We did not use healthy controls because they would not be taking stroke prevention drugs and would not have the same VRF profile. We excluded patients with MRI contraindications, other major neurologic conditions, and severe cardiac and respiratory diseases due to the need to tolerate 6% inhaled CO_2_ during the CVR examination.

After their diagnostic scan, all patients underwent a baseline scanning session within 3 months of the index stroke to avoid any acute effects of the infarct on CVR and other tissue measures. We recorded VRFs, measured blood pressure, and acquired MRI brain images for each patient. Patients returned 1 year after the baseline visit for follow-up MRI scans. At both visits, 2 medical doctors overseen by one expert consultant stroke physician assessed stroke severity and functional outcome using the NIHSS and mRS, respectively. Two researchers assessed cognitive function with the Montreal cognitive assessment (MoCA). Three trained raters overseen by one expert neuroradiologist noted diagnosis of a new stroke/TIA or new infarct on MRI between index stroke and 1-year follow-up.

### MRI Acquisition

During the baseline visit, patients underwent a 1.5-hour MRI scanning session with breaks for patient comfort. We obtained brain images on a 3T MRI scanner (MAGNETOM Prisma, Siemens Healthcare, Erlangen, Germany). Full MRI acquisition protocols have been described previously.^15,16^ Briefly, we acquired 3D T_1_-weighted (T1W; TR/TE/TI = 2500/4.37/1100 ms, flip angle = 7°, 1.0 mm^3^ isotropic resolution), 3D T_2_-weighted (T2W; TR/TE = 3200/408 ms, 0.9 mm^3^ isotropic resolution), 3D fluid-attenuated inversion recovery (FLAIR; TR/TE/TI = 5000/388/1800 ms, 1.0 mm^3^ isotropic resolution), 2D diffusion-weighted gradient-echo echo-planar imaging (EPI) (DWI; TR/TE = 4,300/74 ms, 15 × b_1_ = 0 s/mm^2^, 3 × b_2_ = 200 s/mm^2^, 6 × b_3_ = 500 s/mm^2^, 64 × b_4_ = 1,000 s/mm^2^, 64 × b_5_ = 2,000 s/mm^2^, 2.0 mm^3^ isotropic resolution), and 3D susceptibility-weighted imaging (SWI; TR/TE = 28/20 ms, flip angle = 9^°^, 0.6 × 0.6 × 3.0 mm^3^ resolution) images.^15^ We also acquired 2D gradient-echo EPI images (TR/TE = 1,550/30 ms, flip angle = 67°, 2.5 mm^3^ isotropic resolution) in parallel with a hypercapnic challenge to measure CVR.^16^

We previously established the 6% CO_2_ challenge paradigm to be reliable, reproducible, and well tolerated in older patients and those with recent minor stroke.^7,16^ It is important that this hypercapnic challenge does not only triggers dilation of large arteries, but also arterioles and capillaries in the brain, as evidenced in the retina, which is developmentally related to the brain, where both arterioles and capillaries dilate in response to 6% CO_2_.^6^ The 12-minute gas challenge consisted of breathing medical air and hypercapnic gas (6% CO_2_, 21% O_2_, and 73% N_2_) alternately (in 2-3-2-3-2 minutes cycles) supervised by a physician or nurse.^7^ During the CVR scan, we monitored end-tidal CO_2_ (EtCO_2_), heart rate, respiration rate, and peripheral oxygen saturation level.

At 1-year follow-up, patients returned for repeat structural MRI on the same scanner with the same sequence parameters at baseline (3D T1W, 3D T2W, 3D FLAIR, and 3D SWI). CVR was not measured during this visit.

### MRI Data Processing

Data processing was blinded to clinical information and baseline CVR measurements. Trained raters supervised by an experienced neuroradiologist visually rated SVD features including the number of lacunes and microbleeds using the STRIVE-1 criteria.^17^ One researcher recorded new infarcts after index stroke if visible on DWI or FLAIR images: this information was combined with the recurrence of clinical stroke or TIA to give a binary variable indicating whether the subject had either a new stroke/TIA and/or a new infarct since index stroke.

We registered all structural images to the T2W baseline image space for each patient using FSL FLIRT^18,19^ (FMRIB Software Library, FMRIB Analysis Group, Oxford, United Kingdom). One researcher segmented acute stroke lesions on the FLAIR image under the supervision of an expert neuroradiologist. Using computational methods previously described, we segmented WMH and PVS on the FLAIR and T2W image, respectively.^20–23^ We used the coregistered FLAIR, T1W, and T2W images to generate the brain mask. We segmented NAWM using an in-house developed pipeline that combines FreeSurfer^24,25^ and FSL FAST^26^ outputs. We generated SGM and ventricle masks using Freesurfer.^24,25^ All masks generated by computational pipelines were checked and rectified manually by one researcher where needed. From the generated masks, we extracted multiple volumes. We normalized the WMH and brain volumes to the intracranial volume (ICV) and reported them in %ICV, whereas we normalized the PVS volumes to the volume of the region of interest (ROIV; basal ganglia [BG] or centrum semiovale [CSO]) used for segmentation and reported them in %ROIV units.

**Figure 1:**
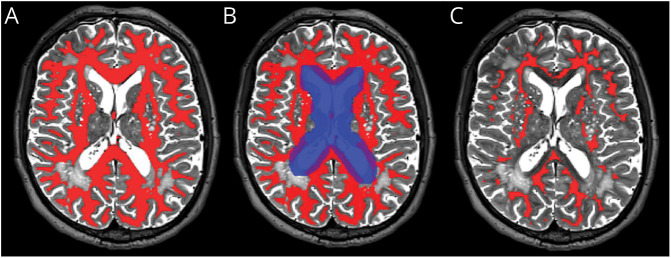
Example of Tissue Processing for the CVR Analysis: NAWM Mask in a Participant's T2W Space (A) NAWM mask (red mask) before processing. (B) The original NAWM mask (red mask) was eroded by 1 mm in all directions, and voxels within the dilated mask of the ventricles (blue mask) were removed. (C) The final NAWM mask (red mask) before registration to the participant's mean BOLD space. BOLD = blood oxygen level-dependent; NAWM = normal-appearing white matter.

One researcher processed the CVR data, which were then checked by another researcher. We eroded the SGM and NAWM masks by 1 mm in all directions in T2W space to reduce partial volume effects (Figure 1). To remove contributions from large blood vessels in tissues adjacent to the lateral ventricles, we dilated the mask of the ventricles by 5 mm left and right and by 4 mm in the anterior, posterior, superior, and inferior directions and, for each patient, subtracted the dilated mask from the NAWM and WMH masks (Figure 1). We further corrected the SGM, NAWM, and WMH masks, if needed, for overlap with large venous blood vessels present on SWI images. We then transformed them into the mean BOLD space using FSL FLIRT after temporally realigning the BOLD images. We used linear regression to model the mean BOLD signal in each ROI. The regressors were a time-shifted EtCO_2_ profile and a linear drift (i.e., volume number).^7^ The optimal delay for the EtCO_2_ profile was defined as the shift resulting in lowest sum of squared residuals. CVR was then defined as the regression coefficient associated with the EtCO_2_ divided by the baseline BOLD signal (i.e., mean BOLD intensity across the first 30 volumes of medical air block) multiplied by 100. Therefore, CVR corresponded to the relative change in BOLD signal per unit change in EtCO_2_ and was reported in units of %/mm Hg.

### Statistical Analysis

We conducted statistical analyses using *R* (version 3.6.1) with the additional packages: tidyverse, ggplot2, insight, sjPlot, and car.

We modeled associations with CVR separately for each tissue (CVR in SGM, NAWM, and WMH). We used multivariable linear regressions for analyses related to quantitative neuroimaging features. In all models, the variable of interest at 1-year follow-up was defined as the outcome and CVR at baseline as an independent variable (univariate analyses in eTable 1). We also adjusted the linear regressions for the variable of interest at baseline (e.g., for brain volume at baseline in the brain volume model) to control for baseline SVD burden (analyses in eTable 2). Furthermore, we adjusted the models for age, sex, mean arterial pressure (MAP), smoking history (current or recent vs ex-smoker for more than 1 year vs never), and hypertension, diabetes, and hypercholesterolemia diagnosis. We used ordinal logistic regression for analyses with 1-year NIHSS, mRS, and MoCA score as outcome, also adjusting for baseline score, age, sex, and VRFs. Finally, we used binomial logistic regression for analyses related to new stroke/TIA diagnosis and appearance of new infarcts on brain scans. We corrected those models for WMH volume at baseline, age, sex, MAP, and VRFs. To avoid overfitting the binomial logistic regressions, we used a score combining VRFs with equal weights (hypertension, diabetes, hypercholesterolemia, and smoking history).^27^

For each model, we checked model assumptions (e.g., normality of residuals and heteroscedasticity), as well as the absence of collinearity between independent variables. To ensure normality of residuals, we transformed the WMH and PVS volumes using the logarithm to the base-10 function. We also conducted several sensitivity analyses to investigate the effect of missing data (eTables 3–5) or to ensure the stability of the results for different technical aspects (i.e., adjusting for the resting EtCO_2_ level, adjusting for baseline WMH volume, and including only CVR data sets of high quality; eTables 6–8).

We excluded data sets with missing baseline or follow-up time points from relevant analyses. We reported regression coefficients or odds ratios along with 95% CIs and *p* Values.

### Standard Protocol Approvals, Registrations, and Patient Consents

All participants gave written informed consent. The study was approved by the Southeast Scotland Regional Ethics Committee (ref. 18/SS/0044) and conducted according to the principles expressed in the Declaration of Helsinki.

### Data Availability

Data will be made openly available when the study has completed; in the meantime, researchers wishing to access MSS3 data can approach the Chief Investigator (Joanna M. Wardlaw).

## Results

**Figure 2:**
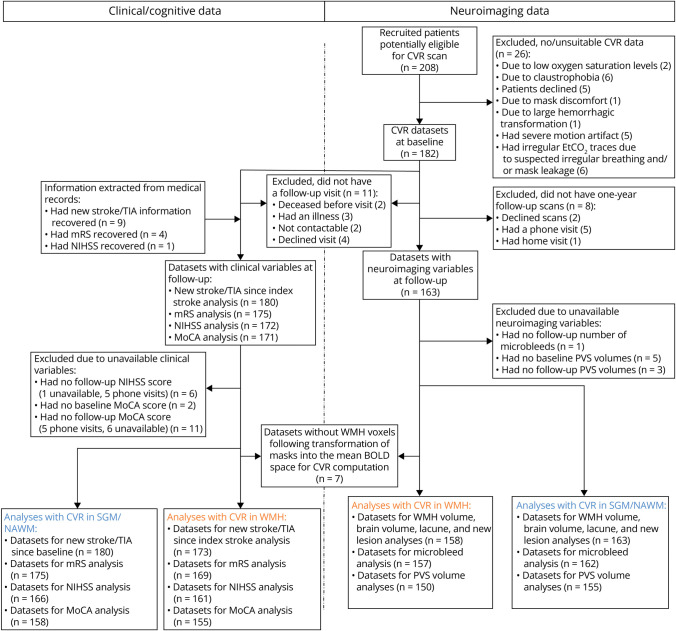
Flow Diagram of Study Recruitment, Data Collection, and Analysis The blue numbers correspond to number of data sets related to CVR analyses in SGM and NAWM, whereas the yellow ones correspond to number of data sets related to CVR analyses in WMH. BOLD = blood oxygen level-dependent; CVR = cerebrovascular reactivity; EtCO_2_ = end-tidal CO_2_; MoCA; Montreal Cognitive Assessment; mRS = modified Rankin score; NAWM = normal-appearing white matter; NIHSS = NIH Stroke Scale; PVS = perivascular space; SGM = subcortical gray matter; WMH = white matter hyperintensity.

We recruited 208 patients of whom 15 did not undergo baseline CVR scanning for medical reasons; 182 of 193 patients had CVR for analyses (Figure 2). One hundred sixty-three of 182 patients (median age: 68.0 years old, 68% male) returned for structural MRI 1 year later with a median number of days between visits of 369 (Figure 2 and Table 1). Sample size, which differed between analyses due to data availability, and reasons for data exclusion are summarized in Figure 2.

**Table 1:** Study Population With Analyzable Baseline CVR and Follow-up MRI Scan (N = 163)

	Baseline	Change after 1 y
Median number of days between index stroke and baseline visit	63 (45, 77)	—
Median number of days between baseline and follow-up visits	369 (360, 387)	—
Median age in years	68.0 (56.2, 74.4)	—
Number of men and women (model denominator: female)	111 (68%), 52 (32%)	—
Median MAP in mm Hg	105 (98,115)	—
Number of diabetes diagnosis (model denominator: no)	32 (20%)	—
Number of hypertension diagnosis (model denominator: no)	118 (72%)	—
Number of hypercholesterolemia diagnosis (model denominator: no)	118 (72%)	—
Number of smokers (current, ever, never; model denominator: never)	26 (16%), 63 (39%), 74 (45%)	—
Number of lacune strokes, number of cortical strokes	66 (40%), 97 (60%)	—
Median WMH volume in mL	8.04 (3.90, 18.89)	0.32 (−0.48, 1.78)
Median WMH volume in %ICV	0.506 (0.242, 1.140)	0.019 (−0.029, 0.117)
Median number of lacunes	1 (0, 3)	0 (0, 0)
Median number of microbleeds	0 (0, 0)	0 (0, 0)
Median brain volume in ml	1,089 (1,009, 1,179)	−15 (−27, −5)
Median brain volume in %ICV	67.6 (64.5, 71.1)	−0.97 (−1.60, −0.34)
Median BG PVS volume in %ROIV	4.89 (3.33, 6.07)	0.24 (−0.34, 0.95)
Median CSO PVS volume in %ROIV	3.31 (2.18, 5.22)	0.32 (−0.12, 1.18)
Median total PVS volume in %ROIV	3.66 (2.47, 5.41)	0.36 (−0.12, 1.15)
Median NIHSS	1 (0, 2)	0 (−1, 0)
Median modified Rankin Scale	1 (1, 1)	0 (−1, 0)
Median MoCA	25 (23, 27)	1 (−1, 3)
Number and of patients with recurrent stroke/TIA or new infarcts between index stroke and 1-y follow-up visit	47 (29%)	—
Median CVR in SGM in %/mm Hg	0.171 (0.135, 0.207)	—
Median CVR in NAWM in %/mm Hg	0.042 (0.033, 0.054)	—
Median CVR in WMH in %/mm Hg	0.040 (0.025, 0.065)	—

Abbreviations: BG = basal ganglia; CSO = centrum semiovale; CVR = cerebrovascular reactivity; ICV = intracranial volume; MAP = mean arterial pressure; MoCA = Montreal Cognitive Assessment; NAWM = normal-appearing white matter; NIHSS = NIH Stroke Scale; PVS = perivascular space; ROIV = volume of region-of-interest; SGM = subcortical gray matter; WMH = white matter hyperintensity.

Demographic, clinical, and neuroimaging variables are given in each row. The value of the variable at baseline is shown in the second column: nonbinary variables are reported as median (Q1, Q3) and binary and smoking variables as number (%). The change in the relevant variables after 1 year is given in the last column as median (Q1, Q3). The denominator of the binary and categorical variables, which are as independent variables in the linear regression models, are emphasized.

Baseline characteristics are given in Table 1. The median number of days between index stroke and baseline visit was of 63 with (Q1, Q3) = (45, 77) (eFigure 1). The median age was 68.0 (56.2, 74.4) years, and the median WMH volume was 8.04 (3.90, 18.89) mL. At follow-up, the 163 patients had a median change of 0.32 (−0.48, 1.78) mL in WMH volume, of −15 (−27, −5) mL in brain volume, and of + 0.36 (−0.12, 1.15) %ROIV in total (i.e., basal ganglia [BG] + CSO) PVS volume. There were no changes in the median number of lacunes and microbleeds. Of 163 patients, 29% had a recurrent stroke or TIA or incident infarct on MRI since index stroke. Population characteristics for each analysis are given in eTable 9.

**Figure 3:**
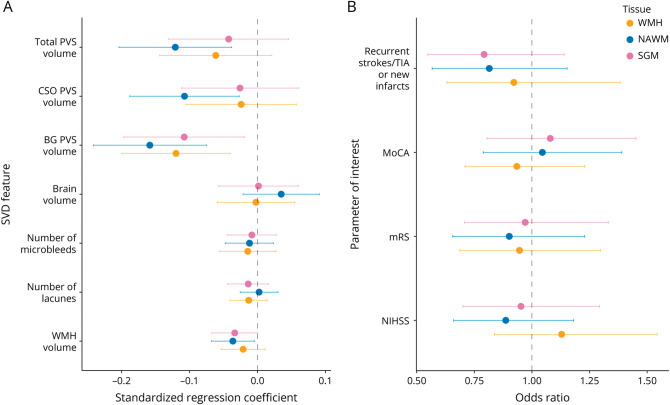
Standardized Regression Coefficients and Odds Ratios Between Parameter of Interest at 1-Year Follow-Up and Baseline CVR (A) Standardized regression coefficients between CVR and quantitative SVD features. (B) Odds ratios between CVR and stroke severity, cognition, recurrent strokes, and new infarcts. CVR was computed in SGM (pink), NAWM (blue), and WMH (yellow). All models were adjusted for the corresponding parameter of interest at baseline (except for new strokes/lesions), age, sex, and vascular risk factors. Models related to NIHSS, modified Rankin scale, MoCA, and new strokes/lesions were also adjusted for baseline WMH volume. The dots represent the mean standardized coefficients and the horizontal lines the associated 95% CIs. The vertical dashed line emphasizes a zero-valued coefficient for linear models and an odds ratio of 1 for ordinal logistic regressions. BG = basal ganglia; CSO = centrum semiovale; CVR = cerebrovascular reactivity; MoCA = Montreal Cognitive Assessment; mRS = modified Rankin Score; NAWM = normal-appearing white matter; NIHSS = NIH Stroke Scale; PVS = perivascular space; SGM = subcortical gray matter; SVD = small vessel disease; WMH = white matter hyperintensity.

**Figure 4:**
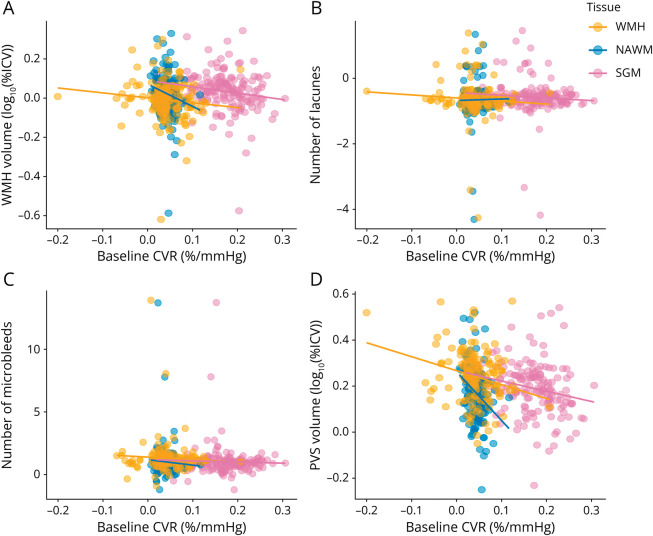
Relationships Between SVD Features After 1 Year and Baseline CVR SVD features shown are (A) WMH volume, (B) number of lacunes, (C) number of microbleeds, and (D) BG PVS volumes. SVD features of interest at 1-year follow-up were adjusted for the corresponding SVD features of interest at baseline, age, sex, and vascular risk factors. The results are shown for adjusted CVR in SGM (pink), NAWM (green), and WMH (blue). The regression lines are shown in the plots. BG = basal ganglia; CVR = cerebrovascular reactivity; NAWM = normal-appearing white matter; PVS = perivascular space; SGM = subcortical gray matter; SVD = small vessel disease; WMH = white matter hyperintensity.

Regression coefficients from the fully adjusted analyses between the parameter of interest at 1-year follow-up and baseline CVR are reported in Table 2 and shown in Figures 3 and 4. We found that lower baseline CVR in NAWM was associated with increased WMH volume after 1 year, with a similar trend for WMH CVR. Lower baseline CVR in all 3 tissues was also associated with increased BG PVS volume after 1 year. In addition, lower baseline CVR in NAWM was associated with increased CSO and total PVS volumes after 1 year. There were no significant associations between baseline CVR and the progression of microbleeds, lacunes, change in brain volumes, NIHSS scores, recurrent stroke or incident infarct on MRI, mRS scores, or MoCA scores.

**Table 2:** Associations Between Baseline CVR in Deep Brain Structures and 1-Year SVD Features (Neuroimaging Features and Clinical Outcomes) Adjusted for the Corresponding SVD Feature at Baseline, Age, Sex, and VRF

Variables after 1 y	SGM CVR	NAWM CVR	WMH CVR	Units of B
WMH volume	B = −0.325 (−0.653 to 0.002) *p* = 0.052	B = −1.14 (−2.13 to −0.14) *p* = 0.026	B = −0.251 (−0.635 to 0.133) *p* = 0.199	log_10_(%ICV) per %/mm Hg
Number of lacunes	B = −0.809 (−2.591 to 0.974) *p* = 0.371	B = 0.454 (−4.852 to 5.761) *p* = 0.866	B = −0.932 (−2.929 to 1.065) *p* = 0.358	Lacunes per %/mm Hg
Number of microbleeds	B = −0.946 (−5.121 to 3.230) *p* = 0.655	B = −4.35 (−17.48 to 8.79) *p* = 0.514	B = −2.01 (−7.85 to 3.82) *p* = 0.496	Microbleeds per %/mm Hg
Brain volume	B = 0.128 (−5.028 to 5.283) *p* = 0.961	B = 9.85 (−6.00 to 25.70) *p* = 0.221	B = −0.241 (−6.320 to 5.839) *p* = 0.938	%ICV per %/mm Hg
BG PVS volume	B = −0.446 (−0.814 to −0.078) *p* = 0.018	B = −2.12 (−3.23 to −1.01) *p* < 0.001	B = −0.607 (−1.010 to −0.203) *p* = 0.003	log_10_(%ROIV) per %/mm Hg
CSO PVS volume	B = −0.143 (−0.620 to 0.334) *p* = 0.554	B = −1.93 (−3.37 to −0.48) *p* = 0.009	B = −0.163 (−0.714 to 0.388) *p* = 0.560	log_10_(%ROIV) per %/mm Hg
Total PVS volume	B = −0.207 (−0.637 to 0.223) *p* = 0.343	B = −1.90 (−3.21 to −0.60) *p* = 0.005	B = −0.366 (−0.858 to 0.126) *p* = 0.144	log_10_(%ROIV) per %/mm Hg
NIHSS	OR = 0.394 (0.001 to 133.151) *p* = 0.753	OR = 0.000609 (0.000000 to 27,725.085042) *p* = 0.416	OR = 16.8 (0.0 to 24,483.1) *p* = 0.431	Per %/mm Hg
mRS	OR = 0.568 (0.001 to 231.960) *p* = 0.853	OR = 0.00173 (0.00000 to 317,342.23552) *p* = 0.517	OR = 0.275 (0.000 to 423.546) *p* = 0.728	Per %/mm Hg
MoCA	OR = 4.29 (0.02 to 1,207.03) *p* = 0.610	OR = 15.2 (0.0 to 615,877,145.1) *p* = 0.758	OR = 0.206 (0.000 to 123.287) *p* = 0.624	Per %/mm Hg
Recurrent strokes/TIA or new lesions	OR = 0.0119 (0.0000 to 12.1976) *p* = 0.210	OR = 0.000003 (0.000000 to 6,079.633,921) *p* = 0.256	OR = 0.149 (0.000 to 1892.095) *p* = 0.681	Per %/mm Hg

Abbreviations: BG = basal ganglia; CSO = centrum semiovale; CVR = cerebrovascular reactivity; ICV = intracranial volume; mRS = modified Rankin Score; MoCA = Montreal Cognitive Assessment; NAWM = normal-appearing white matter; NIHSS = NIH Stroke Scale; PVS = perivascular space; ROIV = volume of region-of-interest; SGM = subcortical gray matter; SVD = small vessel disease; VRF = vascular risk factors; WMH = white matter hyperintensity.

Each row represents a different statistical model where the parameter of interest at 1-year follow-up is given in the first column. The associated regression coefficient B or odds ratio, 95% CI, and *p* value are given in columns 2–4. The last column gives the units of B. All models were corrected for the corresponding parameter of interest at baseline (except for new strokes/lesions), age, sex, and vascular risk factors. Models for NIHSS, mRS, MoCA, and new strokes/lesions were also adjusted for baseline WMH volume to account for SVD burden.

## Discussion

We investigated how baseline CVR is associated with the progression of quantitative SVD features, stroke severity, functional outcome, cognition and stroke/TIA recurrence, and new infarcts on MRI over 1 year in 163 highly phenotyped patients. We found that lower baseline CVR predicted increased WMH and PVS volumes after 1 year, although the strength of this association varied slightly with the tissue type from which CVR and PVS volumes were extracted but were corrected for age, sex, VRFs, and baseline WMH or PVS.

Lower baseline CVR in normal-appearing tissues predicted increased WMH volume after 1 year. A previous study in 45 patients with age-related white matter disease found that CVR in NAWM that progressed into WMH after 1 year was lower than CVR in contralateral NAWM, supporting our findings.^12^ Another study in 25 patients with CADASIL found that lower baseline CVR in the carotid arteries and basilar artery was associated with increased WMH volume after 7 years.^28^ Altogether, the findings suggest that patients with worsening WMH burden over time had lower CVR at baseline. Another study in 60 patients with diverse manifestations of SVD found no significant associations between CVR reduction and progression of a combined score rating the WMH and lacunes after 2 years.^29^ Potential explanations why the results seemingly differ from this study beyond the much smaller sample size, are the following: (1) a combined visual score could be less sensitive to individual SVD neuroimaging features; (2) scores could be less sensitive to subtle changes in neuroimaging features compared with quantitative measurements; and 3) transcranial Doppler ultrasound of CVR in the middle cerebral artery is less sensitive to tissue-level CVR change assessed throughout the subcortical tissues.

We did not find associations between lower baseline CVR and changes in the number of lacunes, microbleeds, or brain volume, in agreement with a previous study (N = 25), which investigated baseline CVR in relation to the 7-year evolution in the number of lacunes and microbleeds.^28^ Overall, this could reflect the limited progression of these SVD features over 1 year, as supported by a previous study,^30,31^ or the lower power to detect changes in these less frequent lesion types. Indeed, most patients had no new lacunes or microbleeds after 1 year: 53% patients had lacunes at baseline and 8% patients had new lacunes at follow-up, 20% patients had microbleeds at baseline and 10% patients had new microbleeds at follow-up, likely not providing sufficient power. We also observed a median brain volume decrease of only of 1% of ICV.

In this study, lower baseline CVR in all ROIs predicted increased BG PVS volume 1 year later. In addition, lower baseline NAWM CVR also predicted 1-year increase in CSO and total PVS volume. We did not consider PVS counts as a marker of SVD progression because the evolution of PVS counts after 1 year could be affected by finite resolution when enlarged PVSs are close in space thereby reducing the number of PVS instead of increasing it, and by the noise in data. Previous cross-sectional analyses (N = 37–53) showed an association between lower CVR and higher PVS scores.^9,31^ The current findings are also in agreement with another longitudinal study in N = 50.^11^ As PVS enlargement is believed to be a marker of impaired waste clearance^32^ and impaired vasomotion,^33^ lower CVR may add to impairment of the brain's drainage system. The association is predominant for BG PVS, perhaps because larger blood vessels are present in the region compared with CSO, thereby making BG more susceptible to changes in vascular fluctuation. It also seems stronger for NAWM CVR, perhaps because SVD affects primarily the white matter (e.g., WMH). However, other SVD features may also contribute to PVS enlargement and overall SVD progression. Therefore, those results should be replicated in future research.

We found that baseline CVR did not predict stroke recurrence or appearance of new infarcts on MRI, possibly reflecting their less frequent occurrence. Instead, future studies could investigate how CVR evolution relates to the appearance of new strokes or infarcts including at a more precise tissue level.

This work has multiple strengths. This is the largest longitudinal study to date to investigate how CVR is associated with the progression of SVD features and clinical outcomes. Moreover, we used a reproducible CVR acquisition and a processing protocol that has been used in multicenter studies^7,16,34^ and image analysis pipelines designed, tested, and optimized for SVD research.^21,22^ Furthermore, 6% CO_2_ affects arterioles and capillaries^6^ and therefore is relevant to detecting vascular changes throughout the subcortical tissues. We included patients with cortical stroke because these control for guideline stroke prevention which all patients received and VRFs which also affect vasoreactivity, and increasing the proportion with fewer SVD features to provide a broad spectrum of SVD severities in the whole sample. Healthy participants would not control for VRFs or stroke prevention drugs and therefore not allow detection of associations specific to SVD.

This work also has limitations. We limited the recruitment to SVD patients with lacunar or mild cortical stroke; therefore, the results might not apply to other forms of SVD (e.g., cognitive presentations). We did not measure interrater reliability, although the ratings of all clinical variables were supervised by one expert and all neuroimaging variables were processed by one researcher per variable type. Missing data over follow-up could have introduced a bias in the analyses; however, given that those missing data could be related to patients' health and would therefore not be missing at random, we could not use statistical methods to correct for them. CVR was only measured at baseline, so we could not observe progression of CVR and SVD features over 1 year or investigate if SVD burden at baseline could predict further CVR impairment. Future studies should consider investigating longitudinal associations over longer periods of time than 1 year. Furthermore, changes in the BOLD signal during hypercapnia were used as a surrogate for changes in cerebral blood flow due to the CO_2_. However, the BOLD contrast is known to also depend on other physiologic variables, for instance the arterial partial pressure of oxygen, hematocrit level or cerebral blood volume, which could have affected the measure of CVR.

In conclusion, lower CVR at baseline predicted an increase in WMH volume and PVS volume after 1 year in SVD patients with mild ischemic stroke, and might be a target for intervention to reduce SVD progression and its clinical outcomes. Further research is needed to understand i) if the reduction of CVR in specific regions is associated with the progression of SVD in the same regions; ii) if the extent of CVR reduction is associated with the burden of SVD progression; iii) the complex relationship between CVR, VRF control, and disease progression; iv) if the findings of the study still hold when considering SVD progression over longer periods; and v) whether SVD damage also precedes further worsening of CVR.

## Appendix Authors

**Table:**

Name	Location	Contribution
Emilie Sleight, PhD	Centre for Clinical Brain Sciences, UK Dementia Research Institute, University of Edinburgh, United Kingdom	Drafting/revision of the manuscript for content, including medical writing for content; major role in the acquisition of data; analysis or interpretation of data
Michael S. Stringer, MSc, PhD	Centre for Clinical Brain Sciences, UK Dementia Research Institute, University of Edinburgh, United Kingdom	Drafting/revision of the manuscript for content, including medical writing for content; major role in the acquisition of data; analysis or interpretation of data
Una Clancy, PhD	Centre for Clinical Brain Sciences, UK Dementia Research Institute, University of Edinburgh, United Kingdom	Drafting/revision of the manuscript for content, including medical writing for content; major role in the acquisition of data; study concept or design; analysis or interpretation of data
Carmen Arteaga-Reyes, MD	Centre for Clinical Brain Sciences, UK Dementia Research Institute, University of Edinburgh, United Kingdom	Drafting/revision of the manuscript for content, including medical writing for content; major role in the acquisition of data; analysis or interpretation of data
Daniela Jaime Garcia, MSc	Centre for Clinical Brain Sciences, UK Dementia Research Institute, University of Edinburgh, United Kingdom	Drafting/revision of the manuscript for content, including medical writing for content; major role in the acquisition of data; analysis or interpretation of data
Angela C.C. Jochems, PhD	Centre for Clinical Brain Sciences, UK Dementia Research Institute, University of Edinburgh, United Kingdom	Drafting/revision of the manuscript for content, including medical writing for content; major role in the acquisition of data; analysis or interpretation of data
Stewart Wiseman, PhD	Centre for Clinical Brain Sciences, UK Dementia Research Institute, University of Edinburgh, United Kingdom	Drafting/revision of the manuscript for content, including medical writing for content; major role in the acquisition of data; analysis or interpretation of data
Maria Valdes Hernandez, PhD	Centre for Clinical Brain Sciences, UK Dementia Research Institute, University of Edinburgh, United Kingdom	Drafting/revision of the manuscript for content, including medical writing for content; analysis or interpretation of data
Francesca M. Chappell, PhD	Centre for Clinical Brain Sciences, UK Dementia Research Institute, University of Edinburgh, United Kingdom	Drafting/revision of the manuscript for content, including medical writing for content; analysis or interpretation of data
Fergus N. Doubal, MD	Centre for Clinical Brain Sciences, UK Dementia Research Institute, University of Edinburgh, United Kingdom	Drafting/revision of the manuscript for content, including medical writing for content; study concept or design
Ian Marshall, PhD	Centre for Clinical Brain Sciences, UK Dementia Research Institute, University of Edinburgh, United Kingdom	Drafting/revision of the manuscript for content, including medical writing for content; analysis or interpretation of data
Michael J. Thrippleton, PhD	Centre for Clinical Brain Sciences, UK Dementia Research Institute and Edinburgh Imaging Facility, Royal Infirmary of Edinburgh, University of Edinburgh, United Kingdom	Drafting/revision of the manuscript for content, including medical writing for content; analysis or interpretation of data
Joanna M. Wardlaw, MD, FRCR, FMedSci	Centre for Clinical Brain Sciences, UK Dementia Research Institute and Edinburgh Imaging Facility, Royal Infirmary of Edinburgh, University of Edinburgh, United Kingdom	Drafting/revision of the manuscript for content, including medical writing for content; study concept or design; analysis or interpretation of data

## Glossary

BG: basal ganglia
BOLD: blood oxygen level-dependent
CSO: centrum semiovale
CVR: cerebrovascular reactivity
EPI: echo-planar imaging
EtCO2: end-tidal CO2
ICV: intracranial volume
MAP: mean arterial pressure
MoCA: Montreal Cognitive Assessment
mRS: modified Rankin Score
NAWM: normal-appearing white matter
NIHSS: NIH Stroke Scale
PVS: perivascular space
SGM: subcortical gray matter
SVD: small vessel disease
VRF: vascular risk factor
WMH: white matter hyperintensities
